# Prevalence of latent tuberculosis infection and associated risk factors in prison in East Wollega Zone of western Ethiopia

**DOI:** 10.1371/journal.pone.0233314

**Published:** 2020-05-19

**Authors:** Basha Chekesa, Balako Gumi, Mahlet Chanyalew, Aboma Zewude, Gobena Ameni

**Affiliations:** 1 Aklilu Lemma Institute of Pathobiology, Addis Ababa University, Addis Ababa, Ethiopia; 2 Collage of Natura and Computational Science, Wollega University, Nekemte, Ethiopia; 3 Ethiopian Public Health Institute, Addis Ababa, Ethiopia; 4 Department of Veterinary Medicine, College of Food and Agriculture, United Arab Emirates University, Al Ain, United Arab Emirates; Jamia Hamdard, INDIA

## Abstract

**Background:**

Latent tuberculosis infection (LTBI) is the major source of active TB and is an obstacle to the strategy of World Health Organization to end TB by 2035. In Ethiopia, there are hundreds of prisons and they are conducive settings for the transmission of TB and could serve as the sources of infection to the general public. However, there is little data on the epidemiology of TB in prisons in Ethiopia. The objective of the present study was to estimate the prevalence of LTBI and evaluate associated risk factors in prisons in East Wollega Zone in western Ethiopia.

**Methods:**

A cross-sectional design and systematic sampling technique were used to select 352 prisoners from a total of 2620 prisoners during the two months (May and June, 2019). The selected inmates were consented for their willingness to participate in the study. Thereafter, they were interviewed and 2ml of blood sample was collected from each prisoner and screened for LTBI using interferon-gamma release assay (IGRA). The data were analyzed using SPSS version 25 and logistic regression was used to model the likelihood of LTBI occurrence and to identify risk factors associated with LTBI.

**Results:**

The prevalence of LTBI was 51.2% (95% CI: 46.45–57%) and higher prevalence was recorded in males (53%) than in females (43.5%) although the difference was not significant. Prisoners whose age ≥45 years (AOR = 2.48, 95%CI, 1.04–5.9), who chewed khat (AOR = 2.27; 95% CI, 1.27–4.19), who were prisoned over a year (AOR = 1.81, 95%CI, 1.04–3.18) and who were in overcrowded pens (AOR = 1.91, 95% CI, 1.002–3.65) were at higher risk of LTBI.

**Conclusions:**

The prevalence of LTBI in prisoners in West Wollega Zone of western Ethiopia was high and could serve as sources of infection to the public. Hence optimum handling of prisoners, and regular follow up and treatment of TB cases in prisons were recommended to minimize the burden of TB in the Zone.

## Background

TB is caused by *Mycobacterium tuberculosis* complex (MTBC) and is spread from person to person via air by droplet nuclei produced when a person with TB coughs, sneezes, talks, or sings therefore causing TB or LTBI [1]. In 2018, there were more than 1.2 million TB deaths worldwide and an additional 251,000 deaths of TB-HIV co-infection. New TB cases reached 10 million and about 3.4% of new TB cases and 18% of previously treated cases had multi-drug resistant TB (MDR-TB) or rifampicin-resistant TB (RR-TB) [2].

LTBI causes persistent immune responses to mycobacterial antigens without evidence of the manifestation of clinical symptoms. It is estimated that approximately two to three billion people living in high TB burden countries are infected with MTBC bacteria [3]. Of those, approximately 1.3 million will develop active TB during their lifetime [4]. Most of these patients develop active TB within the first 5 years unless they are diagnosed and treated with antibiotic drugs [4].

WHO target of elimination of TB by 2050 and End TB Strategy by 2035 (4) could be possible only if the probability of progression LTBI to active TB e is drastically reduced below the current lifetime risk of 5–15% [1,5,6]. In some low-burden countries, reactivation accounts for about 80% of new cases of disease [7]. Hence, in order to reduce the number of new TB cases, WHO adapted from the “End TB Strategy” that states, systematic screening and treatment of LTBI in at-risk populations is a critical component in the elimination of TB [1].

One of the recommended settings to manage LTBI through screening programs is the prison environment [8,9]. This is because that globally, prison represent a major institutional amplifier for TB particularly in low-and middle-income countries (LMICs) [10]. As such, prisons serve as reservoirs that facilitate TB transmission to the general community through released inmates, visitor and prison staff [11]. A systematic review indicated that TB exposure in prisons was attributable to 8.5% and 6.3% of all TB cases in community settings in a high income countries and LMICs, respectively [12]. Additionally, TB contributes significantly to prison related mortality in LMICs [13].

Although, there are global evidences that indicate higher occurrence of TB in prisons as compared to the occurrence of TB in the general population, there are no studies performed on the epidemiology of LTBI in prions in Ethiopia yet and a few studies have been conducted on the epidemiology of TB in prions in Ethiopia [14,15]. These two studies were conducted in the southern and eastern parts of Ethiopia. These studies are not sufficient to represent the epidemiology of TB in prisons in Ethiopia, a country with hundreds of prisons in different regions. Hence, additional studies are needed in order to have national data on the epidemiology of TB in prisons. Particularly, in western Ethiopia, the numbers of prisons and prisoners has rising from time to time since two decades primarily because of political reasons and each prison have been congested with a large number of prisoners. Such situation would favor the transmission of TB leading to high prevalence in the prisons of western Ethiopia. This study was conducted to estimate the prevalence LTBI in prisons in western Ethiopia.

## Materials and methods

### Study settings

A cross-sectional study was carried out in prison located in East Wollega Zonal of western Ethiopia, which is located at about 350 km west of Addis Ababa in Nekemete City. When the study was conducted the study prison was hosting 2620 inmates. Of these inmates 2516 were men, 82 were women and 22 were children. When this study was conducted the Zone had five hospitals, sixty-one health centers and 294 health posts [16].

### Sample size estimation

Sample size was initially estimated using single population proportion formula[17] and using the Formula described by Daniel [18]. In this calculation, 95% confidence interval, 50% margin error and 50% expected prevalence was used. Furthermore, 5% non-respondent was considered and then the final sample size was 352.

### Sampling technique and eligibility criteria

Systematic sampling approach was used to select the study participant from an ordered sampling frame (prisoner). The sampling was started by selecting an element from the list at random and then every *k*^th^ element in the frame was selected [18], where *k*, is the sampling interval, which is every 7 elements was used in this study.

Inmates were eligible for participation into the study if they had no previous treatment for TB; did not have signs and symptoms of TB at the study outset; were above 18-year age and were not pregnant in case of females. Volunteers were informed about the purpose of the study, consented to provide 2ml blood and for their willingness to be interviewed face-to-face for collecting data on risk factors.

### Interferon gamma release assay

Interferon-gamma release assay (IGRA) was performed according to the manufacturer’s instructions (QuantiFERON-TB Gold In-Tube, Cellestis Ldt., Carnegie, Australia). In brief, 2ml of a venous blood was collected from each study participant and 1ml was added into each of the two tubes labelled as ‘**Nil,** negative control and ‘**TB-specific antigens (**ESAT-6, CFP-10, and TB 7.7 antigens), to detect the CD4+ T cell responses to TB antigens. Thereafter, re-mixed by inverting 10 times, and incubated for 24h at 37°C. After incubation the tubes were centrifuged for 15 min at 3000 relative centrifugal force (g) after which the plasma were harvested and stored at − 80°C in Microbiology Laboratory of Wollega University until all samples were collected. After the collection was completed, the plasma samples were transported to Aklilu Lemma Institute of Pathobioogy, Addis Ababa University on ice packs in cold chain and temporarily stored at − 80°C. Thereafter, frozen samples were thawed and used for IGRA. IFN-γ released was measured using the QuantiFERON-TB enzyme linked immunosorbent assay (ELISA) protocol [19]. Sample absorbance was read at a lambda maximum of 450 nm with a reference wavelength of 620 nm using appropriate settings in a 96-well plate spectrophotometer. Results were interpreted as positive, negative or indeterminate with a cut-off value of interferon gamma (IFN-γ) > 0.35 international units per milliliter (IU/ml) using QuantiFERON ®-TB Gold analysis software version 2.62 (Cellestis, Carnegie, Australia) [19].

### Data analysis

Data were entered into epidata and exported to statistical package for the social sciences (SPSS) version 25 software tools. The primary outcome was LTBI status recorded as present or not present, defining a concentration of IFN-γ ≥ 0.35 IU/ml as presence of LTBI. The prevalence of LTBI was estimated by dividing the number of participants with the concentration of IFN-γ ≥ 0.35 IU/ml to the total number of study participants. Frequencies and percentages were used to summarize characteristics of study participants. Bivariate logistic regression analysis was performed to obtain crude odds ratio (OR) with corresponding 95% confidence intervals (95% CI). Multiple logistic regression analysis was performed to assess simultaneously the association between multiple risk factors and the log odds of being positive for LTBI. From this model, adjusted odds ratios (AOR) and 95% CI were obtained.

### Ethical clearance

Ethical approval for the study was obtained from the Addis Ababa University, Aklilu Lemma Institute of Pathobiology Institutional Review Board (ALIPB/IRB/011/2017/2018). Written consent was obtained from each study participant after a clear explanation of the study objectives. Blood samples collection was undertaken after consent was obtained from each participant. Individuals who had LTBI were advised to consult nearby health facilities regarding the development of symptoms of active TB.

## Results

### Socio-demographic characteristics of the study participants

In the study participants (Table 1), 86.9% were male, their age ranged from 18 to 85, mean age ± SD was 27.45 ± 12.6 years and 55.1% of whom were ≤ 24 years. The majorities were unmarried (56.4%), Oromo ethnic group (88.1%) and protestant Christian followers (59.7%). Prior to the beginning of their current sentences, the number of unemployed was 10.8%, while 39.6% and 3.1% were students and housewife respectively, while 66.3% were imprisoned from rural areas, 42.5% were farmers, 41.3% were attending primary school, 24.2% were illiterate 43.4% were married.

**Table 1:** Socio-demographic characteristics of prison inmates (number and frequency) in East Wollega Zone (𝑛 = 352), 2019.

Variable	Response	Number (%)
Sex	Male	306(86.9)
	Female	46(13.1)
Age in years	≤ 24	194(55.1)
	25–44	113(32.1)
	≥45	45(12.8)
Marital status	Single	195(56.4)
	Married	150(43.4)
	Divorced	1(0.2)
Ethnicity	Oromo	309(88.1)
	Amhara	38(10.8)
	Other	4(1.1)
Religion	Protestant Christian	209(59.7)
	Orthodox Christian	107(30.6)
	Muslim	30(8.6)
	Other	4(1.1)
Educational status	Illiterate	85(24.2)
	Primary school	145(41.3)
	Secondary school	101(28.8)
	Tertiary school	20(5.7)
Occupation before imprisonment	Government employed	14(4.0)
	Farmers	149(42.5)
	Self-employed	38(10.8)
	Student	139(39.6)
	House wife	11(3.1)
Residence place before arrested	Rural	232(66.3)
	Urban	118(33.7)
Smoking	Yes	
	No	265(75.5)
Chewing khat	Yes	
	No	259(73.6)
Pre-incarceration alcoholism	Yes	187(53.1)
	No	165(46.9)
Use of inhaled/ injected drugs	Yes	24(7.0)
	No	317(93.0)
Imprisoned previously	Yes	7(2.0)
	No	344(98.0)
Length of incarceration in current prison, months	≤12	200(57.1)
	>12	150(42.9)
Number of prisoners per cell	≤100	96(27.9)
	>100	248(72.1)
Attitude to cell hygiene	Very good	7(2.0)
	Good	106(30.1)
	Bad	125(35.5)
	Very bad	114(32.4)
Sharing of utensils	Yes	255(73.5)
	No	92(26.5)
TB contact history	Yes	217(61.6)
	No	135(38.4)
Place of TB contact	Inside the prison	290(88.1)
	Outside the prison	39(11.9)
Known TB patient in same cell?	Yes	200(58.5)
	No	142(41.5)
Chronically coughing person in same cell?	Yes	235(67.7)
	No	112(32.3)
Health status	Sick	148(46.1)
	Health	173(53.9)
BCG scare	Yes	127(36.3)
	No	223(63.7)
Have you ever homeless before arrested?	Yes	41(12.1)
	No	298(87.9)
Hospitalization history	Yes	61(17.6)
	No	285(82.4)
Currently using antibiotic for any illness	Yes	50(14.5)
	No	295(85.5)
BMI	<18.5	43(12.3)
	18.5–24.99	268(76.8)
	>25	38(10.9)

BMI; Body Mass Index, TB; Tuberculosis; BCG; Bacillus Calmette–Guérin

### Prevalence of LTBI

The prevalence of LTBI at the cut-off point recommended by the manufacturer (≥0.35 IU/ml IFN-γ) in the entire sample was 51.17% (95% CI: 46.45–57%) and with high prevalence in men, rather than women (53.0% vs. 43.5%, respectively; P = 0.231), although no significant difference was highlighted. The prevalence of LTBI increased significantly from 45.5% among those below 24 years to 64.4% in an age ≥45 years (P = 0.024) and among khat chewers than those who were not chewing (63.0% vs 47.7%; p = 0.012).Similarly, the prevalence of LTBI was significantly higher among inmates those imprisoned with >100 individuals than those who imprisoned with <100 individuals (56.5% vs 42.6%; p = 0.022) and among those who stayed long (>1 year) than those who stayed less (≤1 year) (61.1% vs 45.2%; p = 0.004) (Table 2).

**Table 2:** Association between the prevalence of LTBI and risk factors in the prisoners studied bivariate and multivariate analysis (N = 352).

Characteristics	Response	QFT-GIT result		COR (95%CI)	AOR (95%CI)	P value of AOR
		Positive No (%)	Negative No (%)			
Age in years	≤ 24	87(45.5)	104(54.5)	Ref.		
	25–44	64(57.1)	48(42.9)	1.59(0.99–2.55)	1.68(0.93–3.03)	0.083
	≥45	29(64.4)	16(35.6)	2.17(1.11–4.25)	**2.48(1.04–5.9)**	**0.041**
Chewing khat	Yes	58(63.0)	34(37.0)	1.87(1.14–3.05)	**2.27(1.27–4.19)**	**0.009**
	No	122(47.7)	134(52.3)	Ref.		
Length of staying months	≤12	89(45.2)	108(54.8)	Ref.		
	>12	91(61.1)	58(38.9)	1.9(1.23–2.93)	**1.81(1.04–3.18)**	**0.037**
Number of prisoners per cell	≤100	40(42.6)	54(57.4)	Ref.		
	>100	139(56.5)	107(43.5)	1.75(1.08–2.83)	**1.91(1.002–3.65)**	**0.049**
Sharing of utensils	Yes	142(56.3)	110(43.7)	1.8(1.1–2.92)	1.3(0.69–2.46)	0.409
	No	38(41.8)	53(58.2)	Ref.		
TB contact history	Yes	121(56.5)	93(43.5)	1.65(1.07–2.55)	0.89(0.43–1.82)	0.752
	No	59(44.0)	75(56.0)	Ref.		
Place of TB contact	Inside the prison	164(57.1)	123(42.9)	2.04(1.02–4.08)	2.32(0.94–5.75)	0.068
	Outside the prison	15(39.5)	23(60.5)	Ref.		
Sharing cell with TB patient	Yes	112(56.9)	85(43.1)	1.58(1.02–2.45)	1.15(0.57–2.35)	0.695
	No	64(45.4)	77(54.6)	Ref.		
Chronically coughing person in same cell	Yes	130(56.0)	102(44.0)	1.63(1.04–2.58)	1.01(0.52–1.97)	0.979
	No	49(43.8)	63(56.3)	Ref.		
Health status	Sick	83(57.2)	64(42.8)	1.63(1.04–2.54)	1.28(0.74–2.21)	0.381
	Health	78(45.1)	95(54.9)	Ref.		
BMI	<18.5	30(69.8)	13(30.2)	2.56(1.03–6.37)	2.27(0.68–7.59)	0.073
	18.5–24.99	131(49.6)	133(50.4)	1.09(0.55–2.16)	0.82(0.33–2.04)	0.184
	>25	18(47.4)	20(52.6)	Ref.		

LTBI = Latent Tuberculosis Infection; COR = Crude Odds Ratio; ACO = Adjusted Odd Ratio; CI = Confidence Interval; QFT-GIT = Quantiferon®-TB Gold In-Tube; BMI = Body Mass Index

### Factors associated with LTBI

All variables listed in Table 1 were analyzed by bivariate logistic regression and then variables with p < 0.05 were included in multivariable regression analysis and the results of logistic regression analyses were displayed in Table 2. The odds of testing positive were >2 times greater in individuals of age ≥45 years (AOR = 2.48; 95% CI, 1.04–5.9; p = 0.041) compared with younger prisoners. The odds of having LTBI were higher among individuals who chew khat (AOR = 2.27; 95% CI, 1.27–4.19; p = 0.009) compared to those who did not. Similarly, after controlling for other potential confounding variables in the final model, inmates who stayed more than 1 year were nearly 2 times more likely to be QFT-GIT-positive than those who stayed less (AOR = 1.81; 95% CI,1.04–3.18; p = 0.037) and individuals who imprisoned with >100 individuals were also nearly 2 time more likely to be QFT-GIT-positive than those who imprisoned with <100 individuals (AOR = 1.91; 95% CI, 1.002–3.65; p = 0.049) (Table 2).

### IFN-γ response to *Mtb* specific antigens

With regards to the IGRA results, IFN-γ concentration (TB antigen minus the nil) was indeterminate/insufficient in 4(1.13%), negative (<0.20 IU/ml) in 163(46.3%), borderline negative (0.20–0.34 IU/ml) in 5(1.42%), borderline positive (0.35–0.99 IU/ml) in 19(5.39%), in the range of 1–5 IU/ml in 25(7.1%), in the range of 5–10 IU/ml in 9(2.56%) and ≥10 IU/ml in 127(36.08%) for LTBI.

## Discussions

Globally, prisons represent major reservoirs for fueling TB epidemics, particularly in low and middle income countries [20]. The magnitude of LTBI within the Ethiopian prison was remain unknown yet. Thus, this cross-sectional study was to estimate IGRA based prevalence of LTBI and the possible associated risk factors in East Wollega Zonal prisoners. The prevalence observed in this study (51.7%) was different from the prevalence of LTI among the general population as noted by the WHO, which estimates it around 30% [3], as well as by a study carried out in the general Ethiopia population which estimates it around 46% [21] and congruent with pastoral communities in Southern part of Ethiopia (50.5%) [22].

In our findings the prevalence of LTBI were markedly higher than the prevalence in prisons of countries like USA (6.3%) [23]; UK (11.5%) [24], (7.1%) [25]; Australia (14.0%) [26]; Italy (17.9%) [27]; Canada (32.3%) [28]; Spain (40.3%) [29]; Switzerland (46.9%) [30] and Minas Gerais (25.2%) [31]. The probable explanation for this difference might be due to the variation of study population from high-income countries, a prison environment in a country of low TB incidence, more efficient TB control programs both inside and outside prisons and prison-based TB screening programs in these countries than in Ethiopia. However, it was congruent to study done in prison in Nigeria (52.4%) [32] and lower than those prison found in Spain (54.6%) [33]; in three Brazilian prisons: (61.5% [34], 64.1% [35], and 73% [36]); Colombia (67.6%) [37]; Northeastern Malaysia (87.6%) [38]; Malaysian (88.8%) [39]. The possible reasons for the difference might be associated with the variation of the diagnostic methods used, IGRA in our study and previous study were used Tuberculin skin test, the prevalence of LTB infection may be influenced by a prior BCG vaccination, which is known to cause false-positive TST results. Therefore, other studies [25,33,36,39] and this findings are important for World Health Organization’s End TB strategy [1] which requires the treatment of LTBI individuals to meet the reduction targets and strengthens the significance of screening programs on prison entry for TB Control program and thus, we suggest that intervening the progression of LTBI to active TB in prison has remarkable importance for TB prevention and control in TB burden countries.

Several factors create a favorable context for increasing the prevalence of LTBI in prisoners [34,40]. In our study, multivariate analysis showed that an age of ≥45 years, khat chewing, a length of detention and overcrowding were significantly associated with increased odds of being IGRA positivity. In our study, the prevalence of LTBI statistically associated with an aged prisoner that agrees with studies conducted in prisons [25,28,33,41,42]. Similarly, in this study, there was a significant association between the prevalence of LTBI and among khat chewing inmates. This association might be due to, khat is mostly chewed in small overcrowded, unhygienic and unventilated makeshift huts [43], that favors for TB transmission but further studies needed to be verified it. Furthermore, a statistical association between khat chewers and LTBI likely to be due to the influence of khat on the susceptibility to TB infection. Studies were pointing that khat can modulate the host immune response, effect on macrophages. Suppression of immune response usually makes favorable niche for opportunistic infections like HIV and *Mycobacterium* [44–46].

In East Wollega Zonal prison, overcrowding is a real concern that the buildings were purposefully designed to accommodate more than 700 inmates per cell that may enhance the propagation of diseases. In this study, there was statistically significant association between LTBI and the number of inmate per cell (mean per-cell 252.36 ±150.93) and thus, unclogging the prison would lead to greater than 84%-reduction of TB cases and would break the transmission chain between prisoners, staff, and visitors [47], in accordance with reports of Margolis *et al*. and Rueda *et al*. [38,48]. Another factor that has a positive association between the prevalence of LTBI in this prison is how long inmates stayed in prison. Greater length of incarceration has been associated with higher LTBI prevalence in some studies in the literature [27,49], that supports our study.

This study was used an IGRA recommended for the screening of LTBI compared to the skin test. Thus, our data likely reflect the true nature of LTBI prevalence in the entire prison. But there is no “gold standard” for LTBI detection, and the test available is not exempt from errors. The study was also limited by the fact that the subjects were not tested for HIV (we did not obtain ethical approval for HIV testing) which may affect IGRA result.

## Conclusions

The prevalence of LTBI in East Wollega Zonal prisoners were high, being above that of the general population. In this penitentiary facilities, LTBI in is associated to an older age, chewing khat, staying long period in prison and overcrowdings, chewing khat was the variable most strongly associated. Hence, an interventions program, screening and treating inmates with TB infection and disease upon entry into prison should start in Ethiopia prisoners. Number of individuals per cell need to be reduced, prisoners should be counseled and further studies are urgently needed to investigate the prevalence LTBI and associated risk factors in different prisons of Ethiopia.
